# Deacetylated Sialic Acid Sensitizes Lung and Colon Cancers to Novel Cucurbitacin-Inspired Estrone Epidermal Growth Factor Receptor (EGFR) Inhibitor Analogs

**DOI:** 10.3390/molecules28176257

**Published:** 2023-08-26

**Authors:** Mathias T. Anim, Isaac Tuffour, Rylan Willis, Matthew Schell, Trevor Ostlund, Mater H. Mahnashi, Fathi Halaweish, Rachel Willand-Charnley

**Affiliations:** 1 Department of Chemistry and Biochemistry, South Dakota State University, Brookings, SD 57007, USA; aytiem@gmail.com (M.T.A.); isaac.tuffour@jacks.sdstate.edu (I.T.); rylan.willis@jacks.sdstate.edu (R.W.); matthew.schell@jacks.sdstate.edu (M.S.); ostlu104@umn.edu (T.O.); fathi.halaweish@sdstate.edu (F.H.); 2 Department of Pharmaceutical Chemistry, Najran University, Najran P.O. Box 1988, Saudi Arabia; mhmahneshi@nu.edu.sa

**Keywords:** sialic acid, CASD1, apoptosis, estrone analogs, Epidermal Growth Factor Receptor (EGFR)

## Abstract

Cancers utilize sugar residues such as sialic acids (Sia) to improve their ability to survive. Sia presents a variety of functional group alterations, including O-acetylation on the C6 hydroxylated tail. Previously, sialylation has been reported to suppress EGFR activation and increase cancer cell sensitivity to Tyrosine Kinase Inhibitors (TKIs). In this study, we report on the effect of deacetylated Sia on the activity of three novel EGFR-targeting Cucurbitacin-inspired estrone analogs (CIEAs), MMA 294, MMA 321, and MMA 320, in lung and colon cancer cells. Acetylation was modulated by the removal of Sialate O-Acetyltransferase, also known as CAS1 Domain-containing protein (CASD1) gene via CRISPR-Cas9 gene editing. Using a variety of cell-based approaches including MTT cell viability assay, flow cytometry, immunofluorescence assay and in-cell ELISA we observed that deacetylated Sia-expressing knockout cells (1.24–6.49 μM) were highly sensitive to all CIEAs compared with the control cells (8.82–20.97 μM). Apoptosis and varied stage cell cycle arrest (G0/G1 and G2/M) were elucidated as mechanistic modes of action of the CIEAs. Further studies implicated overexpression of CIEAs’ cognate protein target, phosphorylated EGFR, in the chemosensitivity of the deacetylated Sia-expressing knockout cells. This observation correlated with significantly decreased levels of key downstream proteins (phosphorylated ERK and mTOR) of the EGFR pathway in knockout cells compared with controls when treated with CIEAs. Collectively, our findings indicate that Sia deacetylation renders lung and colon cancer cells susceptible to EGFR therapeutics and provide insights for future therapeutic interventions.

## 1. Introduction

**Figure 1:**
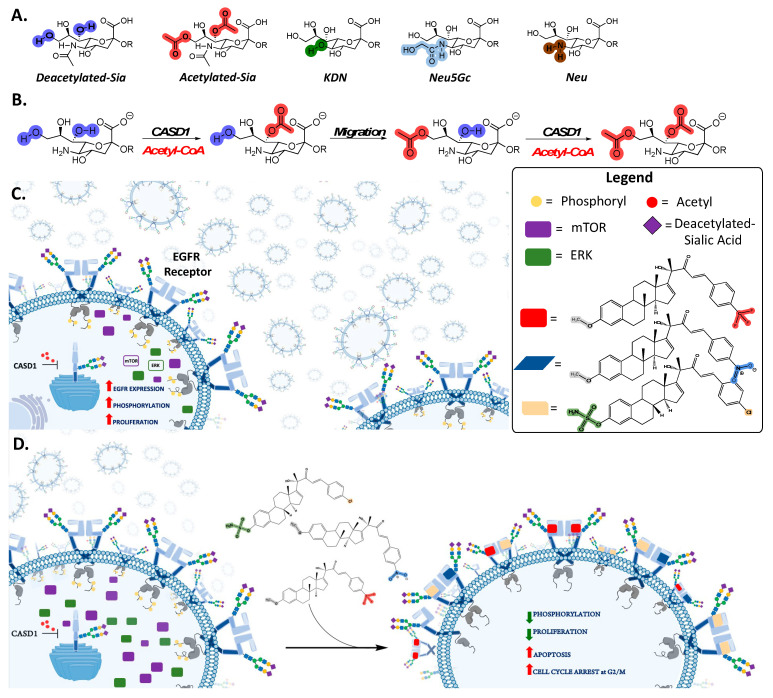
(**A**) Non-canonical forms of Sia (Deacetyl Sia, Acetyl Sia, KDN = Deaminated Sia and Neu5Gc = N-Glycolyl Neuraminic acid) implicated in several biological processes. (**B**) CASD1 enzyme mediates 9-O and 7,9-O acetylation of Sia (Neu 5Ac). CASD1 adds acetyl functional groups, via an Acetyl CoA donor, to the seventh carbon of Sia, from which it migrates to the ninth carbon (Neu 5,9Ac2) under physiological conditions. (**C**) Absence of CASD1 abrogates the addition of acetyl groups to Sia residues on the EGFR glycan chain, resulting in the overexpression of activated (phosphorylated) EGFR, increased levels of activated downstream proteins such as ERK and mTOR and increased proliferation of cells. (**D**) Treatment of CASD1-deficient/deacetylated Sia-expressing cells with CIEAs (i.e., EGFR inhibitor) inhibits EGFR activation, decreases levels of phosphorylated (activated) downstream proteins (i.e., mTOR and ERK), decreases cell proliferation and induces apoptosis as well as G2/M cell cycle arrest/switch.

Sialic acids (Sia), the terminal residues on glycoproteins and lipids are important sugar residues that play key roles in intercellular communication and migration [1,2,3]. Physiologically, Sias and their non-canonical forms (Figure 1A) can be modified with several functional groups, implicating them in the pathogenesis of several disease conditions, including autoimmune disorders, inflammation, coronary artery disease, influenza infections, SARS-CoV2 infections, and cancer among others [4,5,6,7]. The acetylation of Sia, a biological phenomenon which is mediated by Sialate-O-acetyl transferase (CASD1) enzyme (Figure 1B), for instance, has been reported to affect immune cells’ activity, and growth of tumors [1,8,9]. Specifically, aberrant expression of CASD1 has been reported to slow down apoptosis and promote rapid growth of tumors [10]. Tumors in such scenarios respond poorly to chemotherapy, thus warranting alternative treatment regimens. Targeted therapies tend to be a better treatment alternative when these conventional chemotherapies become ineffective [11].

Cancer targeted therapies employ small molecule inhibitors or monoclonal antibodies to selectively inhibit growth of cancer cells based on their distinctive phenotypes [12]. The Epidermal Growth Factor Receptor (EGFR) pathway remains one of the most successfully and effectively targeted signaling pathways in cancer treatment. EGFR is a heavily glycosylated and sialylated transmembrane receptor tyrosine kinase which is activated by epidermal growth factors (EGF) [13]. Following EGF binding, EGFR undergoes conformational changes that result in receptor dimerization, autophosphorylation, kinase activation cascades and downstream signaling pathways responsible for regulating several cellular processes, including cell growth, differentiation, survival, and apoptosis [14]. Existing conventional EGFR/EGFR pathway-targeting therapeutics include monoclonal antibodies such as cetuximab, panitumumab, zalutumumab, nimotuzumab, and matuzumab, as well as small molecule inhibitors such as sorafenib, gefitinib, erlotinib, afatinib, brigatinib and icotinib [15,16,17]. Barriers to the clinical effectiveness of these aforementioned EGFR-targeting therapeutics abound and include untoward side effects, such as high blood pressure, fatigue, loss of hair, skin problems and diarrhea, and the emergence of resistance due to mutations in the drug targets, thus warranting the need for new and effective therapeutics [18]. In an earlier study, novel estrone analogs (CIEAs) inspired by the structure of Cucurbitacins, a group of naturally occurring triterpenoids isolated from medicinal plants with potent anti-inflammatory and anticancer properties [19,20,21], were synthesized and characterized [22]. These synthesized analogs showed promising anti-proliferative and dual inhibitory activity, targeting the EGFR and MAPK pathways in a panel of cancer cell lines [22]. Sia residues and their functional group alterations are exploited by cancers to perpetuate unique aggressive survival hallmarks such as metastasis and are thus touted as key cellular factors influencing cancer treatment and survival outcomes [23,24,25]. Sialylation of EGFR has been reported to suppress EGFR dimerization and autophosphorylation and sensitizes cancer cells to Tyrosine Kinase Inhibitors [13,26]. However, how Sia acetyl functional groups affect EGFR targeted cancer therapeutics remains unexplored.

Previously, we have shown that deacetylated Sia is exploited by colon and lung cancers to evade natural killer cell-mediated cytotoxicity via the Sia–Siglec pathway and also to confer multidrug resistance (MDR) phenotype via breast cancer resistance protein [1,9]. In this study, we focused our attention on how deacetylated Sia affects the effectiveness of EGFR-targeted therapies. We hypothesized that altering levels of acetyl-Sia may affect cancer cell responses to EGFR-targeted therapeutics. Specifically, we investigated how CASD1 gene knockout (i.e., lack of acetyl Sia) affects the activity of three novel Cucurbitacin-inspired estrone analogs (CIEAs), MMA 320, MMA 321, and MMA 294, in in vitro models of lung and colon cancer cells.

## 2. Results and Discussion

### 2.1. Effects of Deacetylated Sia on Inhibitory Activity of CIEAs

Modulating the expression levels of acetyl groups on Sias has been reported to influence influenza C and D infection [7]. Also, the role deacetylated sialic acids play in helping cancer cells evade NK-mediated cytotoxicity has been demonstrated [1]. In this study, we investigated the impact of deacetylated Sia on cancer cell response to targeted cancer therapy. Small molecules (MMA 294, MMA 320, and MMA 321) that are structural analogs of Cucurbitacin (i.e., CIEAs) and inhibitors of pEGFR were used as drug candidates. Sorafenib, a known targeted therapy and a multi-kinase inhibitor was used as a positive control drug. The MTT (tetrazolium-based) cell viability assay was performed to screen the CIEAs. Following exposure to varying concentrations (0–100 μM) of CIEAs for 48 h, we observed a general inhibitory effect of all CIEAs and Sorafenib (positive control) on the cancer cells (Table 1). Compared with wild type cells, CASD1 knockout HCT 116 cells, for instance, showed ~two-fold sensitivity to MMA 294 (IC_50_ = 4.29 μM), MMA 321 (IC_50_ = 6.17 μM) and ~three-fold sensitivity to MMA 320 (IC_50_ = 3.14 μM). Knockout variants of A549 cells showed relatively much higher sensitivity towards the CIEAs compared with their wild type counterpart. MMA 294 exhibited ~four-fold inhibitory activity against the CASD1 knockout. MMA 321 on the other hand, interestingly recorded ~17-fold inhibitory activity against the CASD1 knockout cell line. 

Our results further suggest that Sia deacetylation stemming from removal of the Sia acetyl group functionalizing enzyme, CASD1, though reported in earlier studies to confer multi-drug resistance (MDR) phenotype via upregulation of breast cancer resistance protein [9] (a drug efflux pump that contributes to multidrug resistance, invasiveness (aggressiveness), and self-renewal in cancers [27]), induces some form of chemo-sensitivity in these tumor cells when challenged with the EGFR-targeted CIEAs. This provides insights into a possible alternative chemotherapeutic approach for combating MDR cancer cells.

### 2.2. Apoptotic Effects of CIEAs

**Figure 2:**
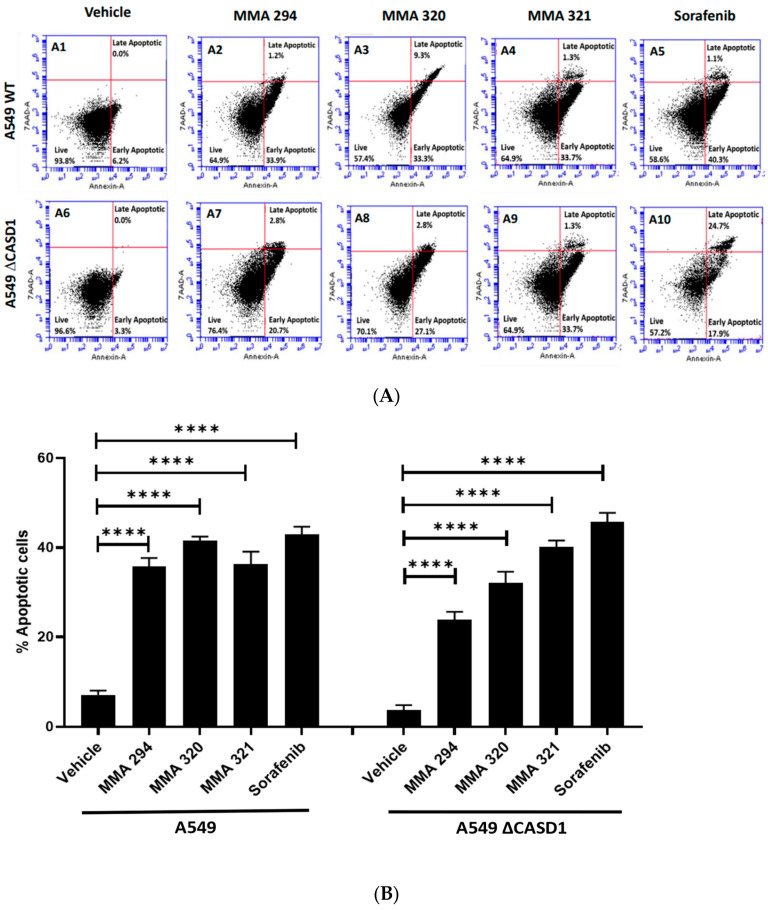
**Apoptotic effects of CIEAs in A549 cells.** Cells were challenged with the CIEAs, harvested, washed, and resuspended in Annexin V binding buffer. Cells were then stained with APC Annexin V and 7-AAD, incubated in the dark for 25 min and analyzed via flow cytometry. (**A**) Flow cytometry histograms of A549 wild type 2A(**A1**–**A5**) and CASD1 knockout 2A(**A6**–**A10**) cell lines. **A1**&**A6**: Vehicle treatment, **A2**&**A7**: MMA 294 treatment, **A3**&**A8**: MMA 320 treatment, **A4**&**A9**: MMA 321 treatment and **A5**&**A10**: Sorafenib treatment (**B**) Comparative apoptosis analysis of A549 cell lines. Two-way ANOVA with Tukey’s post-test was used for multiple comparisons, **** *p* ˂ 0.0001.

**Figure 3:**
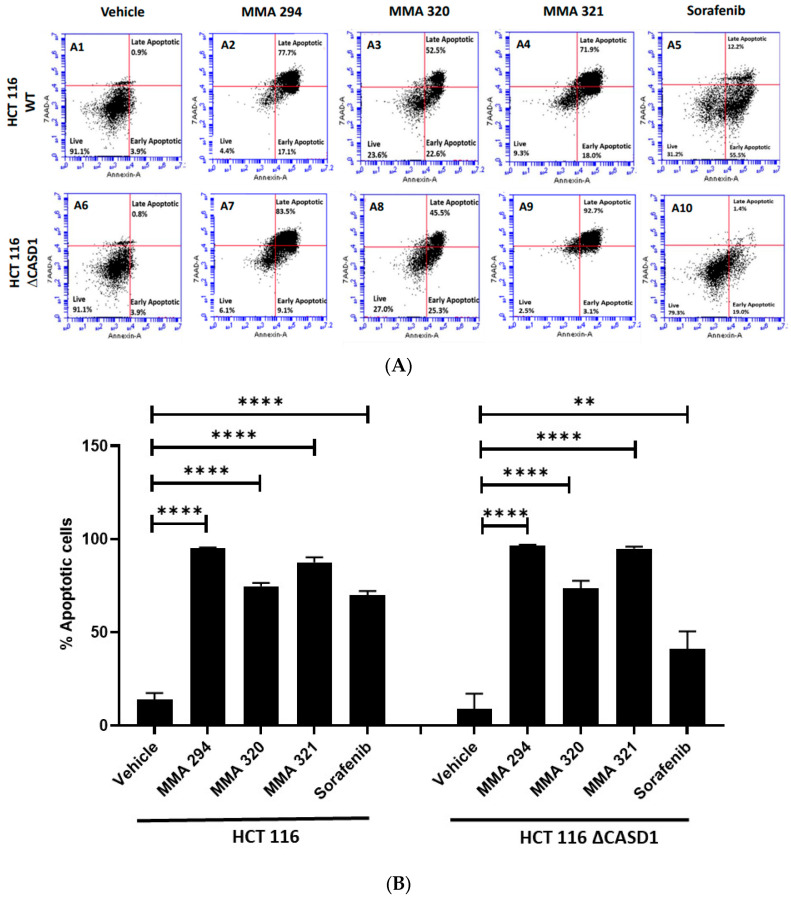
**Apoptotic effects of CIEAs in HCT 116 cells.** Cells were challenged with the CIEAs, harvested, washed, and resuspended in Annexin V binding buffer. Cells were then stained with APC Annexin V and 7-AAD, incubated in the dark for 25 min and analyzed via flow cytometry. (**A**) Flow cytometry histograms of HCT 116 wild type 3A(**A1**–**A5**)) and CASD1 knockout 3A(**A6**–**A10**) cell lines. **A1**&**A6**: Vehicle treatment, **A2**&**A7**: MMA 294 treatment, **A3**&**A8**: MMA 320 treatment, **A4**&**A9**: MMA 321 treatment and **A5**&**A10**: Sorafenib treatment (**B**) Comparative apoptosis analysis of HCT 116 cell lines. Two-way ANOVA with Tukey’s post-test was used for multiple comparisons, ** *p* ˂ 0.01 and **** *p* ˂ 0.0001.

We explored apoptosis as a possible mechanism underlying the observed remarkable growth inhibitory effects of the CIEAs. Apoptosis is a programmed cell death utilized by cells in controlling tissue or organ development as well as cellular turnover [28]. The characteristic features of chromatin condensation, cell volume reduction, endonuclease cleavage of nuclear DNA, loss of nuclear membrane integrity, loss of plasma membrane symmetry and expulsion of phosphatidylserine onto the outer plasma membrane make cells undergoing apoptosis unique [29,30]. In this study, we successfully utilized two of these key apoptotic features in sorting and characterizing CIEA-treated cells to evaluate whether their growth inhibitory effects were via apoptosis. CIEA-challenged cells were further treated with APC-Annexin and 7AAD, two well-known reagents that bind to exposed phosphatidylserine (PS) and nuclear DNA, respectively, to detect cells that are undergoing apoptosis [31]. The mode of cytotoxicity or growth inhibition elicited by MMA 294, 320, 321 and Sorafenib was observed to be apoptosis for both HCT 116 and A549 cell lines, which is characterized by the increase in cells found positive for APC-Annexin V only (lower right quadrant: early apoptosis) or in combination with 7-AAD (upper right quadrant: late apoptosis) (Figure 2A(A1–A10),B and Figure 3A(A1–A10),B). 

These findings confirm and are consistent with previous studies by Mahnashi and colleagues 2019, who reported increased levels of key mediators of apoptosis (i.e., cleaved Caspase 9, cleaved Caspase 3, and cleaved PARP-1) in hepatocellular carcinoma (HepG2) cells when treated with MMA 132, a structural analog of the CIEAs used in this study. In addition, these CIEAs have been shown in earlier studies to be potent EFGR inhibitors, and the inhibition of these EGFR pathways is reported to allow the activation of pro-apoptotic proteins, including Bim, Caspase-9, and Bad [32,33,34], thus promoting apoptosis. MMA 294, MMA 320, and MMA 321 could, therefore, be further explored as potential drug candidates for targeted therapeutics against lung and colon cancers. Comparatively, no significant differences in apoptosis were observed between wild type and CASD1 knockout cells following CIEAs treatment. This suggests that alterations in other cell growth regulatory pathways, including the cell cycle signaling pathway and EGFR signaling pathways, may contribute to the observed sensitivity of knockout cells to the CIEAs compared with wild type cells [35,36].

### 2.3. Effects of CIEAs on the Cell Cycle

**Figure 4:**
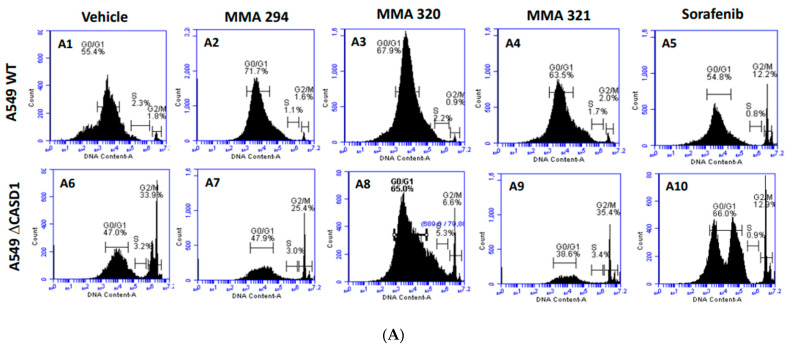

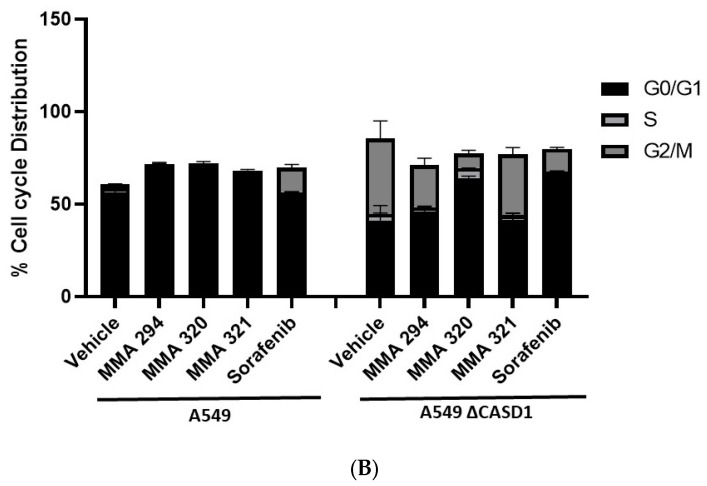
**Effects of CIEAs on A549 Cell Cycle.** Cells were challenged with CIEAs, washed, fixed with 70% ethanol, stained with propidium iodide/RNAse A solution, and analyzed via flow cytometry. (**A**) Flow cytometry histograms of A549 wild type 4A(**A1**–**A5**) and CASD1 knockout 4A(**A6**–**A10**) cell lines. **A1**&**A6**: Vehicle treatment, **A2**&**A7**: MMA 294 treatment, **A3**&**A8**: MMA 320 treatment, **A4**&**A9**: MMA 321 treatment and **A5**&**A10**: Sorafenib treatment (**B**) Comparative cell cycle phase distribution profile of A549 cell lines.

**Figure 5:**
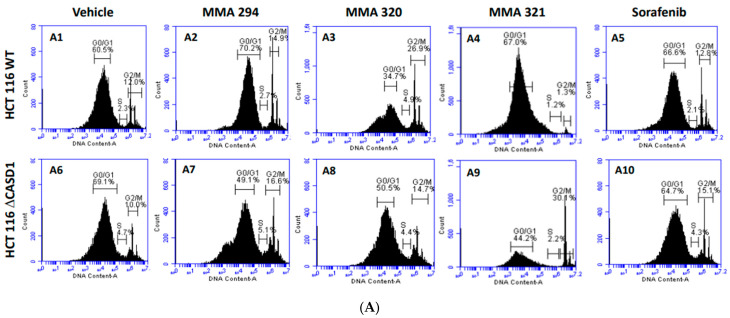

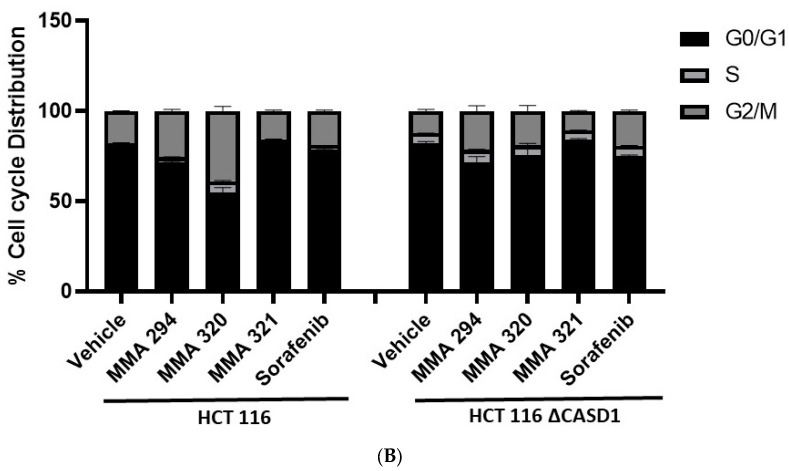
**Effect of CIEAs on HCT 116 Cell Cycle.** Cells were challenged with CIEAs, washed, fixed with 70% ethanol, stained with propidium iodide/RNAse A solution, and analyzed via flow cytometry. (**A**) Flow cytometry histograms of HCT 116 wild type 5A(**A1**–**A5**) and CASD1 knockout 4A(**A6**–**A10**) cell lines. **A1**&**A6**: Vehicle treatment, **A2**&**A7**: MMA 294 treatment, **A3**&**A8**: MMA 320 treatment, **A4**&**A9**: MMA 321 treatment and **A5**&**A10**: Sorafenib treatment (**B**) Comparative cell cycle phase distribution profile of HCT 116 cell lines.

The cell cycle is primarily involved in replication and plays a crucial role in the coordination between cell proliferation and cell death. Cancer cells rapidly replicate and divide by circumventing the tightly regulated cell cycle [37]. Growth factors are critical for the initiation of signaling events that trigger cell cycle progression. In addition to their targeted functions, growth factor/growth factor receptor inhibitors have been reported to dysregulate normal progression of the cell cycle, often resulting in cytostatic or cycle arrest phenomena [38]. Thus, understanding of the coordination between the cell cycle and EGFR inhibitor is crucial to the development of novel and effective cancer therapies. In this study, we elucidated how Sia deacetylation affects the cell cycle (distribution/phases) in HCT 116 and A549 cells following treatment with the CIEAs. The distribution of cells in various phases of the cell cycle (G0/G1, S, G2/M) was observed to be dependent on the type of CIEA and cell type. All the tested CIEAs and Sorafenib were observed to elicit a G0/G1 cell cycle arrest in wild types of both A549 and HCT 116 cells (Figure 4A(A1–A5),B and Figure 5A(A1–A5),B).

Sorafenib in an earlier study was reported to have multiple targets in cells but to arrest HCT 116 and A549 wild type cells at G0/G1 [39,40]. However, irradiation of these same cells before Sorafenib treatment causes a G2/M arrest cell cycle [39], suggesting that altering the genetic composition of a cell could cause changes in how it responds to drugs. Our data suggest that treating CASD1 knockout cells with the same CIEAs causes a switch in cell cycle arrest from the observed G0/G1 in wild type cells to a G2/M cell cycle arrest in A549 cells (Figure 4A(A6–A10),B), corroborating earlier findings. Again, an earlier study revealed that drugs that elicited G2/M arrest did so at concentrations less than those associated with G0/G1 [41]. This may explain why the recorded IC_50_ values were much lower in the knockout cells compared with the wild type cells of both A549 and HCT 116. Also, the G2/M checkpoint prevents cells from entering mitosis when DNA is damaged, providing an opportunity for repair and stopping the proliferation of damaged cells [42]. This may explain the observed decrease in viable cells and increased sensitivity of CASD1 knockout cells relative to wild type cells when challenged with CIEA. 

### 2.4. Effects of Deacetylated Sia on Phosphorylated EGFR Expression

**Figure 6:**
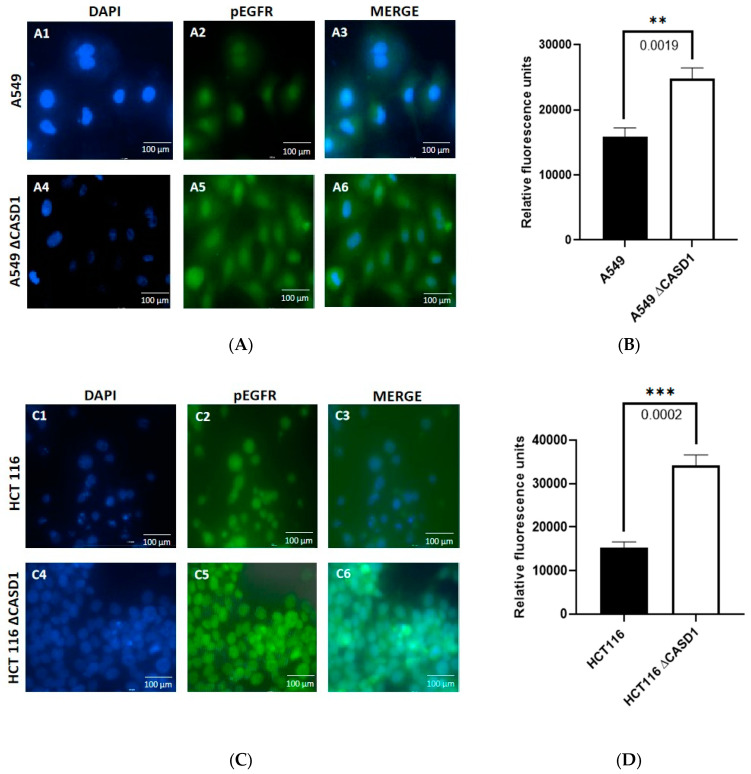
**Effects of Sia deacetylation on Phosphorylated EGFR (pEGFR) expression.** Cells were fixed, permeabilized, treated with pEGFR-specific primary antibody and Alexa-Fluor 488 conjugated secondary antibody. DAPI was used for counter staining. (**A**) Immunofluorescent localization of pEGFR in A549 wild type 6A(**A1**–**A3**) and CASD1 Knockout 6A(**A4**–**A6**) cell lines. **A1**&**A4**: DAPI stained nuclei, **A2**&**A5**: pEGFR fluorescence and **A3**&**A6**: Merged DAPI stained nuclei and fluorescent pEGFR (**B**) pEGFR fluorescence intensity for A549 cell lines. (**C**) Immunofluorescent localization of pEGFR in HCT 116 wild type 6C(**C1**–**C3**) and CASD1 Knockout 6C(**C4**–**C6**) cell lines. **A1**&**A4**: DAPI stained nuclei, **A2**&**A5**: pEGFR fluorescence and **A3**&**A6**: Merged DAPI stained nuclei and fluorescent pEGFR (**D**) pEGFR fluorescence intensity plot for A549 cell lines. One-way ANOVA with Tukey’s post-test was used for multiple plot comparisons, ** *p* ˂ 0.01 and *** *p* ≤ 0.001.

Our data on the viability of the cells following CIEAs treatment revealed increased growth inhibition in the CASD1 knockout HCT 116 and A549 cell lines compared with their wild type counterparts. We, therefore, sought to understand the underlying reason for this observed variation in sensitivities to the CIEAs. CIEAs in earlier in silico and in vitro experimental studies have been reported as potent EGFR inhibitors that reduce levels of pEGFR [22,43,44]. We, therefore, explored levels of activated (pEGFR) as a possible contributory factor. Our immunofluorescence data revealed significantly high expression levels of pEGFR in knockouts of the A549 cell line (*p*-value = 0.0019) (Figure 6A(A5,6),B) and HCT 116 cell line (*p*-value = 0.0002) (Figure 6C(C5,6),D) compared with their wild type cell lines (Figure 6A(A2,3),B,C(C2,3),D). pEGFR overexpression has been reported in several studies as a major factor in poor tumor prognosis. It is associated with a more aggressive clinical progression in several cancers including lung, breast, ovarian, bladder, esophageal and cervical, cancers [45,46,47]. This indicates that CASD1 knockout cells that relatively overexpress pEGFR are a more aggressive cancer type. 

This speculation is consistent with our earlier studies that reported increased cell proliferation in colon and lung cancer cells with deacetylated Sia [7,9]. The findings of this present study, however, show that, this acquired enhanced proliferation trait could also render these deacetylated Sia-expressing CASD1 knockout cells very sensitive to cytotoxicity by pEGFR inhibitors. The increased expression of these growth receptors ensures there are relatively more potential targets for the pEGFR inhibitors (Figure 1C), and this could account for the much lower IC_50_ values recorded in the knockout cells relative to the wild type cells. The removal of acetyl functional groups increases Sialylation (i.e., free Sia) relative to levels of modified Sia; thus, our results are consistent with work conducted by Yen et al., 2015 who reported that Sialylation enhances the sensitivity of resistant lung cancer cells to the EGRF-targeted therapeutic, gefitinib. Our data also supports a previous study that reported a decrease in IC_50_ values in cells that overexpress EGFR when a targeted EFGR chemotherapeutic agent is used [48]. We, therefore, speculate that altered (increased) levels of the protein targets (pEGFR) account for the observed increased susceptibility of knockout cells to CIEAs. The mechanism underlying this observed phenomenon is, however, unknown, and thus warrants further investigation. 

### 2.5. Effects of CIEAs on ERK and mTOR Phosphorylation Levels 

**Figure 7:**
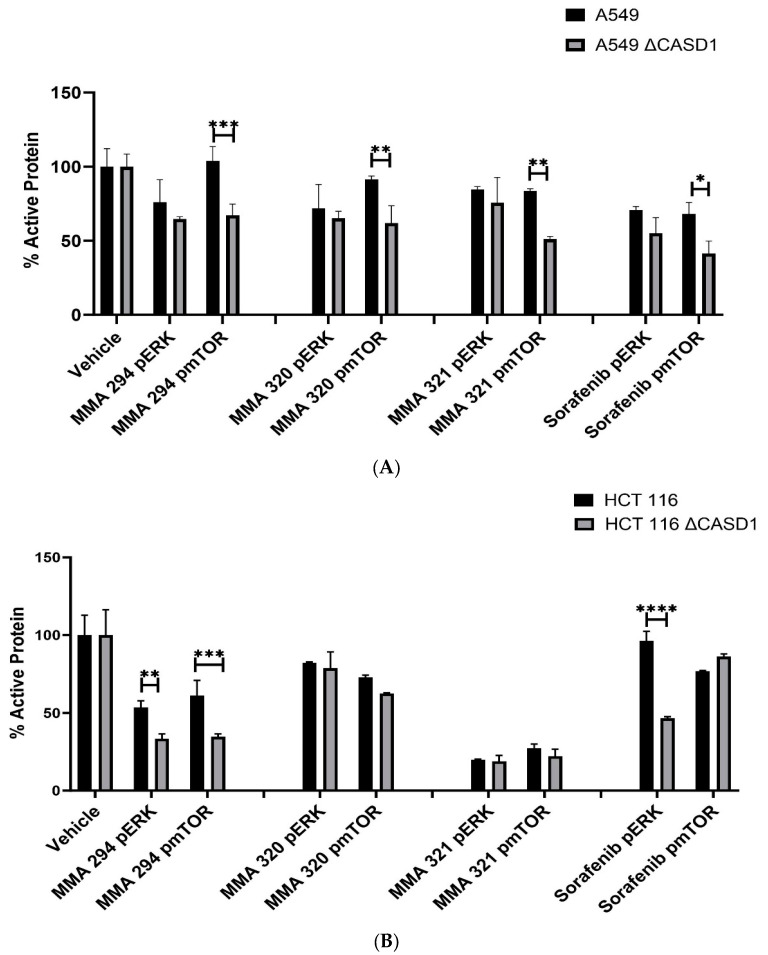
**Effects of CIEAs on phosphorylated ERK and mTOR levels (expressed as percentage of control).** Cells were challenged with CIEAs, fixed, permeabilized, treated with either (p)ERK or (p)mTOR specific primary antibody and goat anti-mouse or anti-rabbit IRDye secondary antibody. (**A**) A549 cell lines. (**B**) HCT 116 cell lines. Two-way ANOVA with Tukey’s post-test was used for multiple comparisons, * *p* ˂ 0.05, ** *p* ˂ 0.01, *** *p* ˂ 0.001 and **** *p* ˂ 0.0001.

To further confirm earlier findings that increased protein target (i.e., pEGFR) renders knockout cells susceptible to the CIEAs relative to wild type cells, we elucidated the effect of CIEAs on the expression of two crucial downstream effectors/proteins of the EGFR signaling pathway: extracellular signal-regulated kinase (ERK) and mammalian target of rapamycin (mTOR). Our results revealed generally reduced levels of active ERK and more significantly reduced active mTOR in knockout cells relative to wild type cells when treated with CIEAs (Figure 7A,B). Compared with wild type cells, MMA 294, MMA 320 and MMA 321 elicited significant decreases in the levels of pmTOR in CASD1 knockout A549 cell lines (*p*-values = 0.0010, 0.0056 and 0.0026, respectively). Sorafenib treatment also showed reduced levels of pERK and pmTOR (*p*-value= 0.0108) (Figure 7A). In HCT 116 cells, MMA 294 elicited a significant reduction in pERK and pmTOR levels in CASD1 knockout cell lines (*p*-values = 0.0063 and 0.0007, respectively) (Figure 7B). The sorafenib treatment resulted in significant decreases in pERK levels in CASD1 knockout cells (*p*-value ≤ 0.0001).

The EGFR signaling pathway is one of the most conserved and well-studied pathways in eukaryotes. It basically entails the transmission of activating signals to downstream protein kinases following ligand (EGF)–receptor (EGFR) binding/interactions. Ultimately, these activating signals activate genes in the nucleus that are involved in cell proliferation, differentiation, migration and survival [34,38,49]. ERK and mTOR are two of the crucial downstream proteins activated in this signaling cascade. Upon activation by phosphorylation, ERK translocates to the nucleus where it activates several transcription factors, including CREB, Fos and Elk-1, ultimately leading to effector protein synthesis and causing changes in cell proliferation and survival [50]. ERK activation also promotes the development of tumors by phosphorylating the two pro-apoptotic proteins, Bim and Bid, causing the proteasome degradation of Bim and the sequestration of Bad to the phosphoserine-binding proteins, thereby inhibiting apoptosis [51]. mTOR is also involved in multiple signaling pathways in the body including phosphoinositide-3-kinase (PI3K)/AKT and serves as a key mediator in the regulation of cell proliferation, autophagy, immune cell differentiation, tumor metabolism and apoptosis [52]. ERK and mTOR are often activated in tumors and have, thus, become hot targets in anti-tumor therapy research.

Our findings are consistent with earlier studies by Mahnashi and colleagues, 2019 who reported that MMA 132, a structural analog of our CIEAs, exhibited dual inhibitory mechanisms against the phosphorylating pathways of the EGFR and MAPK (RAS/RAF/MEK/ERK) pathways. Also, the observed significant decrease in levels of activated mTOR and partly activated ERK in CASD1 knockouts of A549 and HCT 116 cells as compared with wild type cells, especially for MMA 249, suggests that there may be more protein targets (pEGFR) in these deacetylated Sia-expressing cells available for inhibition by the CIEAs, hence the corresponding reduced levels of downstream activated proteins (i.e., pERK and pmTOR). Furthermore, the observed reductions in levels of these proteins could explain the comparatively reduced viability/proliferation and increased apoptosis observed in CASD1 knockout cells when challenged with CIEAs (Figure 1D).

## 3. Materials and Methods

### 3.1. Scientific Rigor

All reagents, antibodies and cell lines used in this study were selected based on published figures and purchased from companies that provide validation. The experiments were all performed in technical and then biological replicates. The data were analyzed with one- and two-way ANOVA followed by Tukey post-tests for multiple comparisons using GraphPad Prism 9 (San Diego, CA, USA). The data are all presented as mean ± standard deviation, with *p* < 0.05 indicating significance.

### 3.2. Chemicals and Reagents 

Tetrazolium dye 3-(4,5-dimethylthiazol-2-yl)-2,5-diphenyltetrazolium bromide (MTT), Alexa Fluor 488 conjugated goat anti-mouse IgG cross-adsorbed secondary antibody (2 mg/mL, Cat #A32723) and paraformaldehyde were obtained from Thermo Fisher Scientific Inc. (Rockford, IL, USA). Phosphorylated EGFR monoclonal IgG1 mouse antibody (15A2:sc-81488) was obtained from Santa Cruz Biotechnology (Dallas, TX, USA). Annexin V binding buffer 1×, APC Annexin V, 7-Amino-Actinomycin D (7-AAD) and PI/RNase staining buffer were obtained from BD Biosciences (San Jose, CA, USA). The secondary goat anti-mouse IRDye secondary antibody was purchased from LI-COR Biotechnologies (Lincoln, NE, USA), and the Vectashield Antifade Mounting medium with DAPI (H-1200-10) was purchased from Vector Laboratories (Burlingame, CA, USA). CIEAs were provided by Dr. Halaweish (South Dakota Ste University, Brookings, SD, USA). All other chemicals used were of analytical grade. 

### 3.3. Cell Lines and Cell Culture

The A549 and HCT 116 cells were grown in Dulbecco modified Eagle medium (DMEM) (Corning) and RPMI 1640 medium, respectively. The media were supplemented with 10% fetal bovine serum (Corning) and 1% pen/strep (Cytiva Hyclone). All the cell lines were originally purchased from the American Type Culture Collection (Rockville, MD, USA). Cell dissociation buffer (Gibco, Waltham, MA, USA) was used exclusively to passage cells. The CASD1 knockout HCT 116 and A549 cell lines were obtained from the group of Colin Parrish (Cornell University). CRISPR-Cas9 editing of CASD1 in HCT116 and A549 cells was previously published [1,7]. Briefly, paired Cas9 plasmids targeting adjacent sites in early exons of CASD1 were transfected using TransIT-X2 (Mirus Bio LLC., Madison, WI, USA). Transfected cells were selected with puromycin, and single cell clones screened with PToV-P4 HE-Fc and sequence-verified to confirm loss of CASD1 function in both alleles [53]. qPCR was also performed to confirm deletion of the CASD1 gene. 

### 3.4. Cell Viability Assay 

The sensitivity of wild type and knockout cells to CIEAs was evaluated using the MTT (3-(4,5-dimethylthiazolyl)-2,5-diphenyltetrazolium bromide) colorimetric assay as previously described [9,54]. Briefly, cells were plated at a density of 2 × 10^5^ cells/mL in a 96-well plate (Corning, Corning, NY, USA) and treated with varying concentrations (0–100µM) of CIEAs for 48 h at 37 °C. MTT solution (5 mg/mL) was then added to each well and cells were further incubated for 4 h at 37 °C. When formazan crystals formed, they were then dissolved by adding stopping solution (Acidified 15% SDS). Absorbance was then measured at 570 nm with a microplate reader (BioTek Cytation 3, Charlotte, WA, USA) and used to estimate the percentage cell viability as shown below. GraphPad Prism 8 software was used to evaluate the individual IC_50_ values.

$$\%Cell\;viability=\left(\frac{Absorbance\;of\;treated\;cells-Absorbance\;of\;blank}{Absorbance\;of\;untreated\;cells-Absorbance\;of\;blank}\right)\times 100$$

### 3.5. Apoptosis Assay

The apoptosis-inducing effects of the CIEAs were evaluated using flow cytometry as previously described [55]. Briefly, cells were plated at a density of 1 × 10^5^ cells/well in a 6-well plate (Corning, NY, USA) and challenged with IC_50_ concentrations of the individual CIEAs for 48 h at 37 °C. The cells were then harvested, washed three times with 1× PBS (Corning, NY, USA) and resuspended in Annexin V binding buffer 1× (BD Biosciences, San Jose, CA, USA). The cells were then stained with APC Annexin V (BD, USA) and 7-AAD (BD Biosciences, San Jose, CA, USA), incubated in the dark for 25 min and analyzed with an Accuri C6 Pus flow cytometer (BD, Biosciences, San Jose, CA, USA). 

### 3.6. Cell Cycle Analysis

Cell cycle analysis was performed on wild type and knockout cells as described previously [56]. In brief, cells (1 × 10^5^ cells/dish) were plated in 6 cm dish. Following treatment with the IC_50_ concentration of CIEAs for 48 h, the cells were centrifuged and washed twice with an assay buffer. The cells were then fixed with 70% ethanol, suspended with staining solution containing propidium iodide (PI) and RNase A, and incubated at room temperature in the dark for 15 min. Analysis was performed within an hour with an Accuri C6 Pus flow cytometer (BD Biosciences, San Jose, CA, USA).

### 3.7. Immunofluorescence Assay

Phosphorylated EGFR expression levels were determined by immunofluorescence microscopy as previously described [9]. Briefly, cells (5 × 10^5^ cells/mL) were cultured on a glass coverslip. The cells were then fixed in 4% paraformaldehyde and permeabilized with 0.5% Triton X-100. The slides were blocked in a buffer containing 0.01% goat serum, 0.01% saponin, and 0.05% glycine in PBS for 1 h and incubated with human phosphorylated EGFR monoclonal IgG1 mouse antibody (Santa Cruz Biotechnology, Dallas, TX, USA) overnight at 4⁰C. The cells were then incubated with Alexa Fluor 488 conjugated goat anti-mouse IgG cross-adsorbed secondary antibody (ThermoFisher Scientific, Waltham, IL, USA) in the dark for 1 h. Vectashield Antifade Mounting medium with DAPI (Burlingame, CA, USA) was placed on each coverslip and inverted on a microscope. A BioTek Cytation 3 Live Cell imager (BioTek, WA, USA) was used to collect immunofluorescence images. 

### 3.8. In-Cell ELISA

The effect of CIEAs on levels of phosphorylated mTOR and ERK was evaluated using the in-cell ELISA method as described previously [56]. Briefly, cells (2 × 10^5^ cells/mL) were plated in 96-well plate and treated with IC_50_ concentrations of CIEAs for 24 h. The cells were then fixed in 3.7% paraformaldehyde and permeabilized with 0.5% Triton X-100. The plates were then blocked with 1x fish gel PBS for 1 h at room temperature and incubated overnight at 4 °C with individual antibodies for ERK, pERK, mTOR, pmTOR and GAPDH (Santa Cruz Biotechnology, CA) at specific dilutions in blocking buffer. Secondary antibody incubation (1:1000 dilution in PBS containing 0.1%Tween 20) was performed using anti-mouse IRDye secondary antibody (LiCOR, Lincoln, NE, USA) and goat anti-rabbit IRDye secondary antibody (Li-COR, Lincoln, NE, USA) 1 h at room temperature. The target proteins were detected using the Odyssey Fc Imager (LiCOR, Lincoln, NE, USA). Fluorescence quantification was performed with Image StudioLite (LI-COR, Lincoln, NE, USA) software and levels of the target proteins (pERK and pmTOR) were normalized and expressed as a percentage of controls.

## 4. Conclusions

The therapeutic limitations and adverse side effects associated with conventional cancer chemotherapies have placed a high demand on targeted therapeutics due to their ability to target cancer cells specifically and effectively. In this study, we have reported on the possibility of enhancing the effectiveness of targeted therapeutics by selectively abrogating the function of a second unrelated protein. More specifically, when Sia acetyl modifying protein, CASD1, is knocked out via CRISPR Cas9 genome editing, lung and colon cancer cells tend to upregulate their activated EGFR expression levels to compensate for the genetic aberration. This deacetylated Sia phenotype causes cancers to be more vulnerable to EGFR inhibitors (CIEAs and Sorafenib) and selectively provides an improved alternative approach through which they can be targeted and killed. Thus, a multidrug targeted therapy that exploits Sia acetyl group inhibitors (or acetyltransferase inhibitors) and EGFR inhibitors could be explored in cancer treatment. Subsequent studies involving animal models are, however, required to further confirm the pre-clinical relevance of these findings.

## Figures and Tables

**Table 1:** Inhibitory effects of CIEAs on HCT116 and A549 cells.

Compound	IC_50_ (µM)	
	A549 WT	A549 ΔCASD1
MMA 294	19.07 ± 5.01	4.77 ± 2.60 (4.0)
MMA 320	9.02 ± 2.32	6.49 ± 1.67 (1.4)
MMA 321	20.97 ± 5.19	1.24 ± 0.09 (16.9)
Sorafenib	26.74 ± 4.38	9.96 ± 3.41 (2.7)
	**HCT 116 WT**	**HCT 116 ΔCASD1**
MMA 294	8.82 ± 0.87	4.29 ± 0.16 (2.1)
MMA 320	8.65 ± 1.14	3.14 ± 0.08 (2.8)
MMA 321	12.72 ± 1.07	6.17 ± 1.74 (2.1)
Sorafenib	9.66 ± 1.58	7.79 ± 0.38 (1.2)

Mean ± SD of three independent experiments performed in triplicate. Fold sensitivity (in brackets) is determined by dividing IC_50_ value of wild type cells by the IC_50_ value of knockout cells.

## Data Availability

The data presented in this study are openly available in Figshare at DOI: 10.6084/m9.figshare.24025542.

## Abbreviations

**CASD1**—CAS1 domain containing protein 1 (Sialate O-acetyltransferase), **CIEAs**—Cucurbitacin inspired estrone analogs, **CRISPR Cas 9**—Clustered regularly interspaced short palindromic repeat-associated protein 9, **EGFR**—Epidermal growth factor, **ERK**—Extracellular signal regulates kinase, **mTOR**—Mammalian target of rapamycin.
